# Osteogenesis imperfecta: the audiological phenotype lacks correlation with the genotype

**DOI:** 10.1186/1750-1172-6-88

**Published:** 2011-12-29

**Authors:** Freya KR Swinnen, Paul J Coucke, Anne M De Paepe, Sofie Symoens, Fransiska Malfait, Filomena V Gentile, Luca Sangiorgi, Patrizia D'Eufemia, Mauro Celli, Ton JTM Garretsen, Cor WRJ Cremers, Ingeborg JM Dhooge, Els MR De Leenheer

**Affiliations:** 1 Department of Otorhinolaryngology, Ghent University Hospital, De Pintelaan 185, B-9000 Ghent, Belgium; 2 Center for Medical Genetics, Ghent University Hospital, De Pintelaan 185, B-9000 Ghent, Belgium; 3 Department of Medical Genetics and Rare Orthopaedic Diseases, Rizzoli Orthopaedic Institute, Via di Barbiano 1/10, I-40136 Bologna, Italy; 4 Department of Pediatrics, La Sapienza University of Rome, Via Regina Elena 324, I-00161Rome, Italy; 5 Department of Otorhinolaryngology, Medical Centre Alkmaar, Wilhelminalaan 12, NL-1815 JD Alkmaar, The Netherlands; 6 FC Donders Institute for Neurosciences, Radboud University Nijmegen Medical Centre, Department of Otorhinolaryngology, PO Box 9101, NL-6500 HB Nijmegen, The Netherlands

**Keywords:** Osteogenesis Imperfecta, *COL1A1*, *COL1A2*, hearing loss, genotype-phenotype correlation

## Abstract

**Background:**

Osteogenesis Imperfecta (OI) is a heritable connective tissue disorder mainly caused by mutations in the genes *COL1A1 *and *COL1A2 *and is associated with hearing loss in approximately half of the cases. The hearing impairment usually starts between the second and fourth decade of life as a conductive hearing loss, frequently evolving to mixed hearing loss thereafter. A minority of patients develop pure sensorineural hearing loss. The interindividual variability in the audiological characteristics of the hearing loss is unexplained.

**Methods:**

With the purpose of evaluating inter- and intrafamilial variability, hearing was thorougly examined in 184 OI patients (type I: 154; type III: 4; type IV: 26), aged 3-89 years, with a mutation in either *COL1A1 *or *COL1A2 *and originating from 89 different families. Due to the adult onset of hearing loss in OI, correlations between the presence and/or characteristics of the hearing loss and the underlying mutation were investigated in a subsample of 114 OI patients from 64 different families who were older than 40 years of age or had developed hearing loss before the age of 40.

**Results:**

Hearing loss was diagnosed in 48.4% of the total sample of OI ears with increasing prevalence in the older age groups. The predominant type was a mixed hearing loss (27.5%). A minority presented a pure conductive (8.4%) or pure sensorineural (12.5%) loss. In the subsample of 114 OI subjects, no association was found between the nature of the mutation in *COL1A1 *or *COL1A2 *genes and the occurrence, type or severity of hearing loss. Relatives originating from the same family differed in audiological features, which may partially be attributed to their dissimilar age.

**Conclusions:**

Our study confirms that hearing loss in OI shows a strong intrafamilial variability. Additional modifications in other genes are assumed to be responsible for the expression of hearing loss in OI.

## 1. Background

Osteogenesis imperfecta (OI) is a hereditary connective tissue disorder, clinically characterized by a variable degree of bone fragility with recurrent fractures, scoliosis, bone deformities, and short stature as well as non-skeletal abnormalities including blue sclerae, abnormal dentition, and joint hyperlaxity. Hearing loss is a common problem in patients with OI caused by type I (pro)collagen defects. On the basis of clinical and radiographic features, Sillence and colleagues [1] distinguished 4 OI subtypes, each representing a different phenotype with a mild (I), lethal (II), severe (III), and moderate (IV) severity. In most cases, the genotype is characterized by a heterozygous mutation in either the *COL1A1 *or *COL1A2 *gene. More uncommon, autosomal recessive forms caused by mutations in other genes have recently been discovered, but have not been associated with hearing loss. The genes *COL1A1 *and *COL1A2 *are both responsible for the formation of type I collagen, the major structural protein of bone, sclerae, ligaments, and tendons. Different mutation types may lead to various OI phenotypes. Nonsense, frameshift, and some splice site mutations result in a reduced synthesis of structurally normal type I collagen, also referred to as haploinsufficiency or a quantitative defect, and usually cause milder phenotypes (OI type I). In the more severe OI types (II-IV), mutations affect the structure of either the proα1(I) or proα2(I) chain and lead to the production of qualitatively abnormal collagen molecules. Glycine substitutions in the triple helical domain are the most common, but some splice site mutations leading to in-frame exon skips may also induce structurally impaired type I collagen. This group of mutations has a negative dominant effect. The overall clinical outcome depends on the type of impairment of type I collagen synthesis (quantitative or qualitative), the mutated gene, the substituting amino acid, and the location of the mutation in the triple helical domain [2,3]. However, genotype-phenotype correlation studies have not been straightforward and inter- and intrafamilial variability in clinical characteristics partially remain unresolved [4].

The variability of the OI phenotype is also observed for the hearing loss and has long been a matter of study and debate. A variable prevalence of hearing loss in OI from 37 to 64% has been reported in family studies [5-11] and from 45 to 58% in nationwide population studies with systematic audiological evaluation [12-15]. In general, hearing loss in OI is believed to develop bilaterally as mild conductive hearing loss in the second to fourth decade of life, subsequently progressing to a mixed hearing loss with a severity ranging from mild to profound [8,10,13-17] Pure sensorineural hearing loss has been observed in a minority of OI patients.[8,13,15-17]. Like the majority of the characteristics inherent to the disease, OI-related hearing loss is apparently heterogeneous in occurrence, type, progression, severity, and underlying pathology. Although it has been suspected to be associated with a *COL1A1 *mutation [18] and to predominantly occur in the milder OI types [1,19], no association has been demonstrated between the presence and type of hearing loss, and the OI subtype, the mutated gene or mutation type in a Finnish population of 47 unrelated OI patients with identified mutations in *COL1A1 *or *COL1A2*[20].

In the present study, audiological and genotypic characteristics as well as potential environmental risk factors for the development of hearing loss are investigated in a cohort of Belgian, Dutch, and Italian OI patients with familial or sporadic OI. In addition, inter- and intrafamilial variability in audiological features are studied.

## 2. Methods

### 2.1 Study subjects

In the period 2008 - 2010, a total number of 184 (89 male; 95 female) of whom 86 Belgian, 67 Dutch, and 31 Italian OI patients participated in this study. They originated from 89 independent families (43 Belgian; 25 Dutch; 21 Italian). The study was approved by the Ethical Committees of the Ghent University Hospital (UZ Ghent), the Radboud University Nijmegen Medical Centre (UMCN), and the Rizzoli Orthopaedic Institute (ROI) of Bologna. According to the Declaration of Helsinki, informed consent was obtained in each patient prior to participation. In Belgium, patients were recruited at the departments of Otorhinolaryngology, Medical Genetics, Orthopedics, and Rheumatology & Endocrinology of the UZ Ghent and by an advertisement in the periodical distributed by the Belgian patients' association for OI (Zelfhulp Osteogenesis Imperfecta). Dutch and Italian OI patients were informed about this study and invited to participate by their otorhinolaryngologists attached to the UMCN and the pediatric clinic of the La Sapienza University of Rome, respectively. All participants had a clinical diagnosis of OI and were examined at the departments of Otorhinolaryngology of the UZ Ghent, UMCN or the pediatric clinic of the La Sapienza University of Rome.

Audiological and molecular data were obtained in the 184 subjects, all of which are analyzed and presented in this paper. Correlation analysis between audiological phenotype and genotype was, due to the adult onset of hearing loss in OI, which is demonstrated below, performed on a subsample of 114 OI patients, hereinafter called the 'selected sample'. The latter consisted of the normal-hearing OI patients older than 40 years and the patients diagnosed with hearing loss at any age.

### 2.2 History and clinical examination

The patients' medical histories and otological complaints were listed. They were interviewed about medication, noise exposure, family history of OI and hearing loss, subjective onset of hearing loss, use of hearing aids, previous ear surgery, head injury, and middle ear infections.

Micro-otoscopy was performed by an otorhinolaryngologist. The clinical OI subtype was assessed by a geneticist.

### 2.3 Audiometry

Pure-tone audiometry was performed in a double-walled sound-attenuated room. Applying the modified Hughson-Westlake method, air conduction (AC) thresholds were bilaterally determined at octave frequencies 0.25 - 8.0 kHz and at half-octave frequencies 3.0 and 6.0 kHz, as well as bone conduction (BC) thresholds at octave-frequencies 0.25 - 4.0 kHz and half-octave frequency 3.0 kHz (AC 40 Clinical Audiometer, Interacoustics, Assens, Denmark). Masking noise was presented at the contralateral ear through a headphone to assess masked AC and BC thresholds according to Hood's plateau method, when interaural differences in AC or intra-aural air-bone gap (ABG) were beyond 40 dB and 10 dB, respectively.

Normal hearing corresponded to AC thresholds < 15 dB HL averaged over 0.5, 1.0, and 2.0 kHz. Hearing loss was classified as (1) conductive: BC thresholds < 15 dB HL and ABG ≥ 15 dB averaged over 0.5, 1.0, and 2.0 kHz; (2) pure sensorineural: AC thresholds ≥ 15 dB HL and ABG < 15 dB averaged over 0.5, 1.0, and 2.0 kHz or AC thresholds > 30 dB HL averaged over 4.0, 6.0, and 8.0 kHz; and (3) mixed: BC thresholds ≥ 15 dB HL and ABG ≥ 15 dB averaged over 0.5, 1.0, and 2.0 kHz. Hearing loss was substantiated by comparison with the 95^th ^percentile value for sex- and age-related hearing thresholds [21]. Concretely, if application of the above-mentioned definitions pointed toward a hearing loss, the patient's audiogram was compared with the 95^th ^percentile threshold values corresponding to the patient's age and sex. When the AC thresholds of the patient exceeded those corresponding to the 95^th ^percentile values, the presence of hearing loss was confirmed. When the AC thresholds from the patient's audiogram fell within the thresholds corresponding to the 95^th ^percentile normal values, the diagnosis of hearing loss was rejected and the patient was classified as 'normal hearing'.

In patients with previous middle ear surgery or cochlear implantation, the preoperative audiometric data were retrieved and included for analysis instead of their recorded postoperative hearing thresholds.

### 2.4 Molecular-genetic analysis

Genomic deoxyribonucleic acid (DNA) was extracted from ethylenediaminetetra-acetic (EDTA) blood samples obtained by standard procedures in all subjects. The methods applied for mutation screening differed somewhat between the Center for Medical Genetics Ghent (Belgian and Dutch patients) and the ROI (Italian patients). In the Dutch and Belgian probands, mutation screening of the *COL1A1 *gene was initiated by polymerase chain reaction (PCR) amplification of the 52 *COL1A1 *exons, using forward and reverse primers. The PCR products were analyzed by gel electrophoresis using the Labchip GX Caliper, software version 2.1.322.0 (Caliper Life Sciences, Hopkinton, MA). All fragments were directly sequenced using the ABI PRISM 3730XL automated sequencer (Applied Biosystems, Foster city, CA). In the Italian probands, complete mutational screening of *COL1A1 *coding regions and exon-intron junctions was performed on DNA by analyzing samples with denaturing high pressure liquid chromatography (DHPLC) followed by direct sequencing of samples with abnormal elution profile. The coding exons of *COL1A1*, along with exon-intron junctions, were PCR-amplified. The results of amplification and the presence of well-sized PCR reaction products were analyzed by gel electrophoresis and visualized by ethidium bromide staining on 2% agarose gels. DHPLC analysis was carried out using the WAVE DNA Fragment Analysis System 3500HT (Transgenomic, Crewe, UK) equipped with a DNASep column (Transgenomic, Crewe, UK). Amplification products showing abnormal elution profiles were reamplified, purified with ExoSAP-IT (USB, Affymetrix, Santa Clara, CA, USA) and sequenced in both forward and reverse direction using BigDye Terminator chemistry version 3.1 and ABI PRISM 3100 automated DNA sequencer (Applied Biosystems, Foster City, CA, USA). Independently of the sequencing method applied, the obtained sequences for all *COL1A1 *exons were compared to the wild-type sequences [GenBank:NG_007400.1] [22]. If no causal mutation could be detected in any exon of the *COL1A1 *gene, the appropiate procedure of those described above was repeated for the proband's 52 *COL1A2 *exons, using the wild-type reference sequences [GenBank:NG_007405.1][22]. Presence of the mutation identified in a proband was subsequently analyzed in all participating relatives by PCR-amplification and sequencing of the corresponding mutated exon in the *COL1A1 *or *COL1A2 *gene.

The outcomes of any previous biochemical analysis on skin fibroblasts or *COL1A1 *null allele tests were retrieved in the concerned medical center and compared to the currently obtained results from molecular-genetic analysis.

### 2.5 Statistical analysis

All data were entered into SPSS for statistical analysis (SPSS Inc., Chicago, IL, USA). Quantitative parameters were evaluated for normal distribution by means of the Kolmogorov-Smirnov test. Associations between dichotomous variables were verified using Chi-square or Fisher's exact test, if appropriate. Differences between median values of two or more independent, quantitative, non-normally distributed variables were evaluated with the Mann-Whitney and Kruskal-Wallis tests, respectively. A 5% significance level was used throughout all analyses.

## 3. Results

### 3.1 Subjects and demographics

Sixty-eight out of the 89 probands reported a positive family history for OI and from 39 of them, one to maximally six affected relatives participated in the study. A sporadic form of OI was noted in 21 probands.

The majority of the 184 patients were diagnosed as OI type I (83.7%). The remaining patients exhibited OI types III (2.2%) and IV (14.1%). Mean age of the patients at audiometric evaluation was 30.5 years (SD: 17.2; range 3-89 years).

### 3.2 General otologic features and audiometric evaluation

Subjective hearing loss was reported by 50.0% of the 184 participants. Hearing amplification was used by 21.2% of the total sample of patients, of whom 87.2% wore hearing aids, 10.3% was equipped with a cochlear implant, and 2.5% had a bone-anchored hearing aid.

The total sample of 184 participants implied 89 probands. Out of these probands, 66.3% reported a positive family history for hearing loss, which referred to either hearing loss in relatives affected with OI (64.0%) or presbyacusis in unaffected relatives of advanced age (2.3%). In none of them, any other genetic cause for familial hearing loss was suspected.

**Figure 1:**
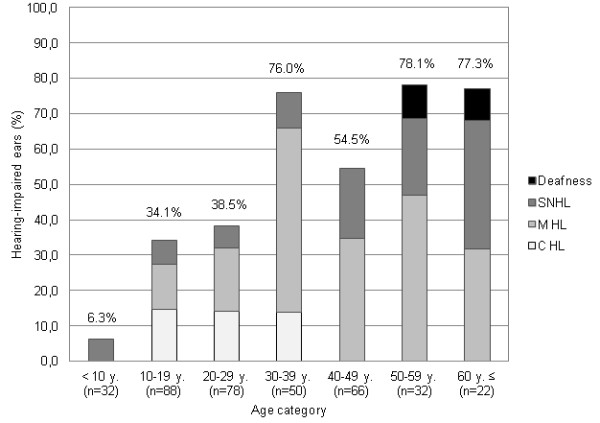
**Prevalence and type of hearing loss as a function of age in 368 ears from 184 osteogenesis imperfecta patients**. The percentages of ears demonstrating conductive hearing loss (C HL), mixed hearing loss (M HL), pure sensorineural hearing loss (SNHL) or total deafness are displayed for seven different age categories. For each age category, the corresponding number of ears is indicated (n).

Audiometric evaluation revealed that 52.7% of the examined OI patients exhibited a unilateral (8.7%) or bilateral (44.0%) hearing loss, resulting into a 48.4% prevalence hearing impairment in the total number of 368 OI ears. Figure 1 presents the proportions of hearing-impaired ears and the different types of hearing loss as a function of age, the latter classified into decades. Five ears of patients in the older age categories demonstrated total deafness. Retrieval of audiometric history revealed that progressive mixed hearing loss had preceded the deafness in all cases.

The overall median age at onset of the hearing loss as reported by the patients was 20 years (range: 5-60 years). Pure sensorineural hearing loss was characterized by a significantly later median onset (36.5 y.; range: 10-60 y.) compared to conductive or mixed hearing loss (19.0 y.; range: 5-42 y.) (Mann-Whitney test; P < 0.001).

In 14.8% of the patients with bilateral hearing loss, audiograms from left and right ears were asymmetric. In the patients with unilateral hearing impairment, pure conductive (n = 4), mixed (n = 4), and pure sensorineural hearing losses (n = 8) were observed.

### 3.3 Environmental risk factors for hearing loss

On the basis of a thorough history, a potential risk for noise-induced hearing loss was noted in a total number of 22 OI patients (12.0%), of whom five demonstrated bilaterally normal hearing. Two patients reporting noise exposure exhibited pure sensorineural hearing loss bilaterally. The remaining 15 patients had conductive or mixed hearing loss.

A total number of nine patients (4.9%) had sustained a skull fracture, one of whom exhibited unilaterally conductive, and three bilaterally mixed hearing loss. The remaining five patients had bilaterally normal hearing. None of the patients with skull fractures attributed development or deterioration of their hearing loss to the trauma.

### 3.4 Molecular-genetic findings

Molecular-genetic screening of the 89 probands identified 69 *COL1A1 *(77.5%) and 20 *COL1A2 *(22.5%) mutations. In the probands with a *COL1A1 *mutation we found nonsense (n = 12), frameshift (n = 29), and splice site (n = 21) mutations, as well as glycine substitutions (n = 7). Splice site mutations (n = 2) and glycine substitutions (n = 18) were observed in the probands with a mutation in the *COL1A2 *gene. The retrieved results of previous biochemical and *COL1A1 *null allele tests carried out on the probands' fibroblasts, confirmed a structural defect of type I collagen for one splice site mutation, and haploinsufficiency of type I collagen for 11 other splice site mutations. The quantitative either qualitative effects of the splice site mutations on type I collagen synthesis was not determined in 10 probands, in whom biochemical tests had not been performed previously and skin fibroblasts were not obtained.

The mutations were confirmed in all the probands' participating affected relatives, resulting in a total number of 150 OI patients with *COL1A1 *(81.5%) and 34 OI patients with *COL1A2 *mutations (18.5%).

### 3.5 Associations between genotype and audiological phenotype in a selected sample of 114 OI patients

For the investigation of potential associations between hearing loss and the underlying genotypic characteristics, only the subjects older than 40 years or those that already had developed hearing loss before 40 years of age were included. This cut-off age was introduced on the basis of the hearing loss onset, which was situated before the age of 40 years in 90.8% of all hearing losses, and in 98.4% of the hearing losses with a conductive component. Two patients meeting these criteria were excluded because of pure sensorineural loss that was probably due to noise exposure. The selected 114 subjects, called 'the selected sample', are listed per family (N = 64) including characteristics of hearing and genotype (see additional file 1: Audiological and molecular-genetic findings in the selected sample of 114 osteogenesis imperfecta patients). The investigation of the intrafamilial variability was based on the 26 families from the 64 families in the selected sample with two or more participating relatives.

#### 3.5.1. Audiological findings in the selected sample

From the 114 patients in the selected sample, only 19 subjects demonstrated bilaterally normal hearing (16.7%). The remaining 95 subjects had hearing loss in one (12.2%) or both ears (71.1%). Of the total number of 228 ears in the selected sample, 54 ears presented normal hearing (23.7%), whereas 31 ears were diagnosed with pure conductive loss (13.6%), 101 ears with mixed hearing loss (44.3%), and 42 with ears pure sensorineural hearing loss (18.4%).

#### 3.5.2. Molecular-genetic findings in the selected sample

A mutation in *COL1A1 *was identified in 51 out of the 64 probands (79.7%) included in the selected sample. The mutational event was located in the *COL1A2 *gene in 13 other probands (20.3%). In 40/64 probands, the mutation caused haploinsufficiency of type I collagen (62.5%), whereas a structural type I collagen defect was encountered in 17 probands (26.6%). In 7 probands affected by splice site mutations (10.9%), the either quantitative or qualitative type I collagen defect was not determined due to the lack of skin fibroblasts and biochemical tests.

#### 3.5.3. Interfamilial variability

In only eight out of the 64 families from the selected sample, none of the participating affected relatives demonstrated hearing loss (12.5%). In six of these families, quantitatively impaired type I collagen synthesis was caused by a mutation in the triple helical domain of the proα1(I) chain, whereas in the other two families a qualitative defect due to a mutation in the triple helical domain of the proα2(I) chain was found. In the remaining 56 families from the selected sample (87.5%), conductive/mixed (44/64 or 68.8%) and/or pure sensorineural hearing loss (20/64 or 31.2%) occurred in at least one affected relative. Conductive/mixed hearing loss occurred in OI families with mutations leading to quantitative type I collagen defect that were located in the N-terminal propeptide (n = 1), C-terminal propeptide (n = 2), and the triple helical domain (n = 25) of the proα1(I) chain, as well as in OI families with qualitatively impaired type I collagen caused by mutations located in the triple helical domain of the proα1(I) chain (n = 5) or the proα2(I) chain (n = 4). In seven families with *COL1A1 *splice site mutations of which the effect on type I collagen was undetermined, conductive/mixed hearing loss was also found. Pure sensorineural hearing loss was observed in families in which OI was associated with haploinsufficiency of type I collagen due to a mutation in the triple helical domain (n = 12) or the C-terminal propeptide (n = 2) of the proα1(I) chain, and in families with a structural type I collagen defect due to a mutation in the triple helical domain of the proα1(I) (n = 1) or proα2(I) chain (n = 5). This hearing loss type was also diagnosed in three families with splice site mutations located in the triple helical domains of the proα1(I) chain (n = 2), of which the type I collagen defect was unknown. Application of Chi-square and Fisher's exact test could not demonstrate that development of hearing loss and the type of hearing loss in OI patients were dependent of the mutated gene, the effect of the mutation on type I collagen synthesis or the location of the mutation within the proα chains.

#### 3.5.4. Intrafamilial variability

Investigation of the intrafamilial variability in 26 families from the selected sample with two to maximally five affected relatives revealed that OI relatives with an identical mutation in *COL1A1 *or *COL1A2 *demonstrated heterogeneous hearing characteristics. In some families, subjects with conductive or mixed hearing loss had relatives with pure sensorineural hearing loss. Additionally, patients with conductive, mixed or pure sensorineural hearing loss had normal-hearing affected relatives. In contrast, in a number of families, certain correspondence was observed with respect to the presence of a conductive or sensorineural component in at least one ear of two or more relatives. The presence of a conductive component in at least one ear of two related subjects was seen in 18/26 families (69.2%) (families Nos. 2, 5, 7, 9, 12, 18, 24, 25, 26, 27, 28, 29, 30, 34, 38, 39, 40, and 54). Likewise, 18/26 families (69.2%) implicated at least two relatives with a sensorineural hearing loss component in one or both ears (families Nos. 2, 5, 7, 8, 9, 12, 15, 20, 25, 26, 27, 28, 29, 30, 37, 38, 39, and 40). Pure sensorineural hearing loss affecting two relatives from the same family was only seen in 4/26 families (15.4%) (families Nos. 8, 20, 26, and 37). A hearing loss with conductive component in all participating relatives was noted in 11 different families. In only one family with two or more participants, all relatives demonstrated bilaterally normal hearing (family No. 10). Furthermore, our selected sample included unrelated families that were sometimes affected by the same mutation. This applied to three different mutations, which were each found in two unrelated families (*COL1A2, c.1009G > A *in families Nos. 1 and 19; *COL1A1, c.1299+1G > A *in families Nos. 44 and 50; and *COL1A1, c.769G > A *in families Nos. 7 and 22). However, the subjects from two families affected by the same mutation demonstrated incongruous audiometric results. In contrast, all relatives from three unrelated families affected by the same underlying *COL1A1 *mutation (*c.769G > A*) were diagnosed with a hearing loss characterized by a conductive component (families Nos. 5, 17, and 54).

#### 3.5.5. Analysis of risk factors for hearing loss in the selected sample

Finally, the selected sample was investigated for associations between hearing loss and positive history for otitis media, skull fracture or noise exposure. Although 25 out 27 of the OI patients with a history of recurrent otitis media currently demonstrated hearing loss (92.6%), the latter was not associated with a positive history of otitis media, because 70 of the 95 hearing-impaired OI patients did not report any episode of otitis media (73.7%) (Chi-square test). Five patients had sustained a skull fracture, of whom one presented bilaterally normal hearing, one unilaterally pure sensorineural loss, and three bilaterally mixed or conductive hearing loss. Noise exposure was confirmed by 14 subjects with mixed hearing loss and three normal-hearing patients. The results of Fisher's exact test were not indicative for significant associations between a history of noise exposure or a skull fracture and the development of hearing loss.

## 4. Discussion

### 4.1 Audiological characteristics

Hearing loss deserves attention in patients with OI, especially since its development, the age at onset, as well as the progression and site of lesion seem difficult to predict on the basis of genotype or hearing characteristics in affected relatives. The prevalence of OI patients affected by hearing loss in the present study amounted to 52.7%. Although a certain selection bias in favour of hearing loss cannot be ruled out, this value approximates the prevalence values established in other family and population studies [5-10,13-15,17,23]. Though, the number of hearing-impaired patients has been assumed to grow with increasing age, reaching almost 100% in the age group older than 60 years in some studies [10,14-16]. We noted that in the patient group over 60 years of age, 77.3% of subjects bilaterally demonstrate hearing thresholds exceeding the 95^th ^percentile of age- and sex-related thresholds in the healthy population, whereas under 30 years of age less than 50% of the OI subjects presented hearing loss.

Most authors agree that the majority of OI patients initially develop a conductive loss in the second to fourth decade of life which gradually progresses to a mixed hearing loss [13-17]. Mixed hearing loss was the predominant hearing loss type in the current study-population. Pure conductive loss only occurred in patients under 40 years of age. This hearing loss has often been attributed to an otosclerosis-like pathology involving stapes footplate fixation and diffuse otosclerotic foci in the temporal bone. In addition, ossicular discontinuity, most often caused by atrophic or fractured crura, may be responsible for conductive hearing loss in OI [10,14,16].

Pure sensorineural loss has been described to occur in smaller but almost constant proportions in all age groups [8,13,15,16]. The sensorineural component in OI has been attributed to atrophy of the cochlear hair cells and the stria vascularis, hemorrhage into the perilymph, microfractures, otosclerotic bone encroaching on the otic capsule, and membrane distortions [11,24].

The hearing loss commonly develops bilaterally and symmetrically, independently of the type of hearing loss involved. Conductive and mixed losses are most often reported to start in the second decade whereas the onset of pure sensorineural loss may be situated at any age.

### 4.2 Associations between hearing loss and genotype

Although hearing loss has been suggested to occur predominantly in OI type I [1], this was not supported by a Finnish population study [13] in which no correlation was found between the OI subtype and the frequency or severity of hearing loss. Our results reflected hearing loss to be a clinical manifestation in OI type I as well as types III and IV. Because of the small number of type III and type IV subjects included, no conclusions can be drawn for associated hearing loss with a specific OI type.

In the study by Hartikka et al.[20], in which audiometric evaluation was accomplished in 49 unrelated OI patients with identified mutations in the *COL1A1 *or *COL1A2 *gene, no correlation was found between the mutated gene, the mutation type or type I collagen defect and the presence, type or severity of hearing loss. Consistently, the present study fails in its effort to interrelate the audiological phenotype from 114 OI patients originating from 64 distinct families with the underlying OI genotype. In 87.5% of the OI families, OI-related hearing loss of the conductive/mixed or pure sensorineural type is observed in at least one relative. Hearing loss affects both patients with familial OI and sporadic OI. We noted intrafamilial variability in hearing characteristics, with relatives sustaining different types of hearing loss. Neither the mutated gene, the quantitatively either qualitatively impaired type I collagen or the location of the mutation with respect to the triple helix appeared to play a role in the expression of hearing loss. Other genetic or environmental factors are likely to contribute to the development of hearing loss and its characteristics in OI.

In an attempt to identify potential risk factors for the development of hearing loss, patients' histories of otitis media, head injury or noise exposure were documented. Despite the higher incidence of otitis media in the OI population reported in the literature, which has been hypothesized to follow from cranial molding and deformities [17,25], a positive history of otitis media was not associated with hearing loss as 73.7% of hearing-impaired patients had not experienced any otitis media episode. Still, about 90% of the OI subjects reporting otitis media in childhood currently presented hearing loss. Neither noise exposure nor head injury was found to be associated with OI-related hearing loss. Head injury or skull fractures were most often reported to have occurred after the hearing loss onset and were also noted in patients without hearing loss. Noise exposure was reported in about 20% of patients with mixed hearing loss and 5% of normal-hearing patients. Because these risk factors were listed on the basis of personal perception by the patients themselves, one may throw doubt upon their relevance. Though, the risk to sustain supplementary damage on hearing due to noise exposure, head trauma or sequelae of otitis media should be reduced to a minimum.

## 5. Conclusions

In this study among 184 Belgian, Dutch, and Italian OI patients, hearing loss presents in 53% of patients and the proportion of hearing-impaired patients rises with increasing age. The predominant type of hearing loss is a bilateral, symmetric, and progressive mixed hearing loss. In a subsample of 114 OI patients with hearing loss or older than 40 years, an attempt to link the audiological phenotype with the underlying OI genotype is performed. The manifestation, type or severity of hearing loss correlates neither with the mutated gene nor with the type I collagen defect or the location of the mutation in the gene. Furthermore, identical mutations are found to lead to considerable inter- and intrafamilial variability in hearing pattern. However, in the present study, inter- and intrafamilial comparisons of hearing characteristics are limitated by the dissimilar age of the subjects and relatives, while hearing loss characteristics may evolve over time. Furthermore, in the majority of the families included, not all affected relatives were examined because they declined to participate or were living abroad. Although history of otitis media, head trauma, and noise exposure were more often reported in the hearing-impaired OI patients, none of these potential risk factors were consistenly associated with the occurrence of hearing loss. In conclusion, the mutation causing OI is not sufficient for the expression of hearing loss. Therefore, an additional genetic trigger is assumed to be responsible for hearing loss in OI.

## Competing interests

The authors declare that they have no competing interests.

## Authors' contributions

FS contacted and invited the Belgian and Dutch patients for participation, performed audiological evaluation and molecular genetic analysis, collected and analyzed all data, and drafted the manuscript. PC substantially contributed to the concept of the study, coordinated the genetic tests, and assisted in structuring the manuscript. ADP assisted in the clinical diagnosis, genetical counseling and critically revised the text. SS supervised the molecular-genetic analysis, checked out the causality of the mutations and formulated valuable suggestions for the text of the manuscript. FM refered and recruted patients, assessed the clinical OI type, offered genetic counseling to the patients and provided valuable insights for data analysis. FG and LS executed molecular-genetic screening and analysis in the Italian patients and wrote out parts of the text. PDE recruted Italian OI patients and subjected them to examination of hearing. TG assisted in the recruitment and hearing evaluation of Dutch patients, and gave critical clues for data analysis. CC substantially contributed to recruitment of Dutch patients, data collection, performed clinical examination and hearing evaluation of patients in the Netherlands, and added critical notes to the manuscript. ID was the supervisor of the study, perfomed otological examination of patients and revised the manuscript. EDL was involved in the delineating the project, otological examination of the Belgian patients and in drafting the manuscript. All authors read and approved the final manuscript.

## Supplementary Material

Additional file 1 **Audiological and molecular-genetic findings in the selected sample of 114 osteogenesis imperfecta patients**. The hearing loss characteristics and results from molecular genetic tests in 114 individuals with osteogenesis imperfecta, grouped per family, are presented. These data reflect inter- and intrafamilial variability in audiological phenotype. Click here for file

## References

[B1] Sillence DO Senn A Danks DM Genetic heterogeneity in osteogenesis imperfecta J Med Genet 1979 16 101 116 10.1136/jmg.16.2.101 PMC1012733 458828

[B2] Byers PH Wallis GA Willing MC Osteogenesis imperfecta: translation of mutation to phenotype J Med Genet 1991 28 433 442 10.1136/jmg.28.7.433 PMC1016951 1895312

[B3] Marini JC Forlino A Cabral WA Barnes AM San Antonio JD Milgrom S Hyland JC Körkko J Prockop DJ De Paepe A Coucke P Symoens S Glorieux FH Roughley PJ Lund AM Kuurila-Svahn K Hartikka H Cohn DH Krakow D Mottes M Schwarze U Chen D Yang K Kuslich C Troendle J Dalgleish R Byers P Consortium for osteogenesis imperfecta mutations in the helical domain of type I collagen: regions rich in lethal mutations align with collagen binding sites for integrins and proteoglycans Hum Mutat 2007 28 209 221 10.1002/humu.20429 PMC4144349 17078022

[B4] Basel D Steiner RD Osteogenesis imperfecta: recent findings shed new light on this once well-understood condition Genet Med 2009 11 375 385 10.1097/GIM.0b013e3181a1ff7b 19533842

[B5] Carey MC Fitzgerald O McKiernan E Osteogenesis imperfecta in twenty-three members of a kindred with heritable features contributed by a non-specific skeletal disorder Q J Med 1968 37 437 449 5676881

[B6] Stoller FM The ear in osteogenesis imperfecta Laryngoscope 1962 72 855 869 10.1288/00005537-196207000-00002 13917432

[B7] Carruth JA Lutman ME Stephens SD An audiological investigation of osteogenesis imperfecta J Laryngol Otol 1978 92 853 860 10.1017/s0022215100086229 712217

[B8] Cox JR Simmons CL Osteogenesis imperfecta and associated hearing loss in five kindreds South Med J 1982 75 1222 1226 10.1097/00007611-198210000-00016 7123292

[B9] Quisling RW Moore GR Jahrsdoerfer RA Cantrell RW Osteogenesis imperfecta. A study of 160 family members Arch Otolaryngol 1979 105 207 211 10.1001/archotol.1979.00790160041011 426710

[B10] Riedner ED Levin LS Holliday MJ Hearing patterns in dominant osteogenesis imperfecta Arch Otolaryngol 1980 106 737 740 10.1001/archotol.1980.00790360015006 7436848

[B11] Shapiro JR Pikus A Weiss G Rowe DW Hearing and middle ear function in osteogenesis imperfecta JAMA 1982 247 2120 2126 7062527

[B12] Kuurila K Hearing loss, balance problems and molecular defects in osteogenesis imperfecta PhD thesis 2003 University of Turku, Department of Otorhinolaryngology

[B13] Kuurila K Kaitila I Johansson R Grenman R Hearing loss in Finnish adults with osteogenesis imperfecta: a nationwide survey Ann Otol Rhinol Laryngol 2002 111 939 946 10.1177/000348940211101014 12389865

[B14] Pedersen U Hearing loss in patients with osteogenesis imperfecta. A clinical and audiological study of 201 patients Scand Audiol 1984 13 67 74 10.3109/01050398409043042 6463554

[B15] Stewart EJ O'Reilly BF A clinical and audiological investigation of osteogenesis imperfecta Clin Otolaryngol Allied Sci 1989 14 509 514 10.1111/j.1365-2273.1989.tb00414.x 2612030

[B16] Garretsen AJ Cremers CW Huygen PL Hearing loss (in nonoperated ears) in relation to age in osteogenesis imperfecta type I Ann Otol Rhinol Laryngol 1997 106 575 582 10.1177/000348949710600709 9228859

[B17] Pillion JP Shapiro J Audiological findings in osteogenesis imperfecta J Am Acad Audiol 2008 19 595 601 10.3766/jaaa.19.8.3 19323351

[B18] Sykes B Ogilvie D Wordsworth P Wallis G Mathew C Beighton P Nicholls A Pope FM Thompson E Tsipouras P Consistent linkage of dominantly inherited osteogenesis imperfecta to the type I collagen loci: COL1A1 and COL1A2 Am J Hum Genet 1990 46 293 307 PMC1684971 1967900

[B19] Paterson CR Monk EA McAllion SJ How common is hearing impairment in osteogenesis imperfecta? J Laryngol Otol 2001 115 280 282 10.1258/0022215011907442 11276328

[B20] Hartikka H Kuurila K Korkko J Kaitila I Grenman R Pynnonen S Hyland JC la-Kokko L Lack of correlation between the type of COL1A1 or COL1A2 mutation and hearing loss in osteogenesis imperfecta patients Hum Mutat 2004 24 147 154 10.1002/humu.20071 15241796

[B21] ISO-7029 Acoustics - Statistical distribution of hearing thresholds as function of age 2000 Geneva, Switzerland, International Organisation for Standardisation

[B22] Dalgleish R A database of osteogenesis imperfecta and type III collagen mutations http://www.le.ac.uk/genetics/collagen

[B23] Garretsen TJ Cremers CW Clinical and genetic aspects in autosomal dominant inherited osteogenesis imperfecta type I Ann N Y Acad Sci 1991 630 240 248 10.1111/j.1749-6632.1991.tb19594.x 1952595

[B24] Berger G Hawke M Johnson A Proops D Histopathology of the temporal bone in osteogenesis imperfecta congenita: a report of 5 cases Laryngoscope 1985 95 193 199 10.1288/00005537-198502000-00014 3918222

[B25] Imani P Vijayasekaran S Lannigan F Is it necessary to screen for hearing loss in the paediatric population with osteogenesis imperfecta? Clin Otolaryngol Allied Sci 2003 28 199 202 10.1046/j.1365-2273.2003.00685.x 12755755

