# Supplementary material for: Osteogenesis imperfecta: the audiological phenotype lacks correlation with the genotype
Source: Orphanet J Rare Dis. 2011 Dec 29;6:88. doi: 10.1186/1750-1172-6-88 (PMC3267664; doi:10.1186/1750-1172-6-88)
Supplement: Additional file 1 — Audiological and molecular-genetic findings in the selected sample of 114 osteogenesis imperfecta patients. The hearing loss characteristics and results from molecular genetic tests in 114 individuals with osteogenesis imperfecta, grouped per family, are presented. These data reflect inter- and intrafamilial variability in audiological phenotype. [file 1750-1172-6-88-S1.PDF]

# Additional file 1

**Audiological and molecular-genetic findings in the selected sample of 114 osteogenesis imperfecta patients**

| Family No. | F/Sp | Individuals | | Hearing characteristics | | | Genotypic characteristics | | | | OI type |
|---|---|---|---|---|---|---|---|---|---|---|---|
| | | M/F | Age | Hearing (L/R) | Onset | Otological history | *COL* | c-notation p-notation | Struct/Haplo | Location | |
| 1 | F | M | 31 | M/M | 25 | NE | *1A2* | *c.1009G>A p.Gly337Ser* | Struct | Triple helix | IV |
| | | M | 43 | 0/0 | | | | | | | |
| | | M | 51 | 0/0 | | NE | | | | | |
| 2 | F | F | 42 | M/M | 22 | NE | *1A1* | *c.3079delG p.Asp1027ThrfsX81* | Haplo | Triple helix | I |
| | | F | 43 | 0/0 | | | | | | | |
| | | F | 65 | M/M | 42 | | | | | | |
| 3 | F | F | 40 | S/0 | 38 | SF | *1A1* | *c.2028+1G>A*[a] | Haplo[b] | Triple helix | I |
| 4 | F | M | 41 | S/S | 20 | OM | *1A2* | *c.2558G>T p.Gly853Val* | Struct | Triple helix | I |
| 5 | F | F | 13 | M/C | 9 | | *1A1* | *c.658C>T p.Arg220X* | Haplo | Triple helix | I |
| | | M | 55 | M/M | 9 | SF | | | | | |
| 6 | F | F | 45 | M/M | 12 | | *1A1* | *c.3258delC*[a] *p.Ala1087ProfsX21* | Haplo | Triple helix | I |
| 7 | F | F | 18 | M/M | 30 | NE | *1A1* | *c.769G>A p.Gly257Arg* | Struct | Triple helix | IV |
| | | M | 38 | M/M | 17 | NE | | | | | |
| 8 | F | F | 36 | S/0 | 35 | | *1A1* | *c.3637delG*[a] *p.Gly1213AlafsX26* | Haplo | C-terminal propeptide | I |
| | | F | 60 | S/S | 50 | | | | | | |
| 9 | F | F | 31 | M/M | 17 | | *1A1* | *c.3765delC*[a] *p.Ala1256ProfsX76* | Haplo | C-terminal propeptide | I |
| | | F | 32 | M/M | 30 | | | | | | |
| | | F | 67 | M/M | 18 | | | | | | |
| 10 | F | M | 40 | 0/0 | | | *1A1* | *c.1792C>T p.Arg598X* | Haplo | Triple helix | I |
| | | M | 41 | 0/0 | | NE | | | | | |
| | | F | 42 | 0/0 | | | | | | | |
| 11 | F | F | 47 | 0/0 | | | *1A1* | *c.3370-1delG*[a] | Haplo[b] | Triple helix | I |
| 12 | F | M | 24 | M/0 | 20 | NE | *1A1* | *c.1128delT p.Gly377AlafsX164* | Haplo | Triple helix | I |
| | | F | 53 | M/M | 39 | NE | | | | | |
| 13 | F | F | 16 | M/M | 14 | | *1A1* | *c.543+2T>C*[a] | Haplo[b] | N-terminal propetide | I |
| 14 | F | F | 40 | 0/0 | | OM | *1A1* | *c.3495delT p.Gly1166ValfsX73* | Haplo | Triple helix | I |

| **Family No.** | **F/Sp** | **Individuals** | | **Hearing characteristics** | | | **Genotypic characteristics** | | | | **OI type** |
|---|---|---|---|---|---|---|---|---|---|---|---|
| | | **M/F** | **Age** | **Hearing (L/R)** | **Onset** | **Otological history** | ***COL*** | **c-notation<br>p-notation** | **Struct/Haplo** | **Location** | |
| 15 | F | F | 22 | M/0 | 21 | | *1A1* | *c.2128-3T>G*[a] | Haplo[b] | Triple helix | I |
| | | F | 49 | M/M | 35 | OM | | | | | |
| 16 | F | F | 40 | M/M | | | *1A2* | *c.2414G>C*<br>*p.Gly805Arg* | Struct | Triple helix | I |
| | | F | 54 | S/S | 49 | | | | | | |
| 17 | F | F | 21 | M/C | 16 | OM | *1A1* | *c.658C>T*<br>*p.Arg220X* | Haplo | Triple helix | I |
| 18 | F | F | 26 | 0/C | 18 | OM | *1A1* | *c.334-9A>G*[a,c] | Unknown | N-terminal propeptide | I |
| | | F | 52 | M/0 | 18 | | | | | | |
| 19 | F | F | 37 | S/S | 25 | | *1A2* | *c.1009G>A*<br>*p.Gly337Ser* | Struct | Triple helix | IV |
| 20 | F | M | 46 | S/S | 42 | | *1A1* | *c.484delC*[a]<br>*p.Gln162SerfsX102* | Haplo | Triple helix | I |
| | | M | 49 | 0/S | 15 | | | | | | |
| | | M | 75 | M/M | 22 | | | | | | |
| 21 | F | M | 30 | M/M | 22 | OM; NE | *1A1* | *c.2016dupC*[a]<br>*p.Ser673LeufsX37* | Haplo | Triple helix | I |
| 22 | F | F | 46 | S/S | 12 | OM | *1A1* | *c.769G>A*<br>*p.Gly257Arg* | Struct | Triple helix | I |
| 23 | F | F | 44 | 0/0 | | | *1A1* | *c.1984-5C>A* | Haplo | Triple helix | I |
| 24 | F | M | 24 | C/C | 16 | | *1A1* | *c.3241delG*<br>*p.Val1081LeufsX27* | Haplo | Triple helix | I |
| | | F | 37 | C/C | 35 | | | | | | |
| | | M | 49 | M/M | 33 | | | | | | |
| | | M | 54 | 0/0 | | | | | | | |
| 25 | F | F | 32 | M/M | 27 | OM | *1A1* | *c.1003-2A>G* | Unknown | Triple helix | I |
| | | M | 60 | S/M | 38 | NE | | | | | |
| 26 | F | M | 16 | S/S | 13 | | *1A1* | *c.579delT*<br>*p.Gly194ValfsX71* | Haplo | Triple helix | I |
| | | M | 45 | M/M | 35 | NE | | | | | |
| | | F | 50 | S/S | 10 | | | | | | |
| | | M | 50 | S/S | 28/40 | | | | | | |
| | | F | 54 | M/M | 15/20 | OM | | | | | |
| 27 | F | M | 16 | M/M | 14 | | *1A1* | *c.2451+94G>T*[c] | Unknown | Triple helix | I |
| | | F | 18 | 0/M | 18 | | | | | | |
| | | M | 49 | 0/0 | | NE | | | | | |

| Family No. | F/Sp | Individuals | | Hearing characteristics | | | Genotypic characteristics | | | | OI type |
|---|---|---|---|---|---|---|---|---|---|---|---|
| | | M/F | Age | Hearing (L/R) | Onset | Otological history | *COL* | c-notation p-notation | Struct/Haplo | Location | |
| 28 | F | F | 21 | M/M | 12 | OM | *1A1* | *c.2559+1G>A*[a] | Haplo[b] | Triple helix | I |
| | | F | 35 | M/M | 13 | | | | | | |
| | | F | 44 | M/M | 11 | OM | | | | | |
| | | M | 50 | M/M | 18 | OM | | | | | |
| 29 | F | M | 36 | M/M | 16 | OM; NE | *1A1* | *c.670G>A*[a] *p.Gly224Ser* | Struct | Triple helix | I |
| | | M | 46 | M/M | 30 | SF | | | | | |
| | | M | 46 | M/M | 15 | OM | | | | | |
| | | F | 54 | M/M | 25 | OM | | | | | |
| | | F | 73 | M/M | 37 | | | | | | |
| 30 | F | M | 19 | C/C | 19/25 | | *1A1* | *c.2028+2T>G* | Haplo | Triple helix | I |
| | | M | 29 | M/M | 18/20 | | | | | | |
| | | M | 39 | M/M | 30 | NE | | | | | |
| | | M | 57 | S/M | 36 | NE | | | | | |
| 31 | F | F | 30 | 0/M | 25 | | *1A1* | *c.3477delT p.Gly1160AlafsX79* | Haplo | Triple helix | I |
| 32 | F | M | 60 | 0/S | 40 | | *1A1* | *c.1668delT p.Gly557ValfsX23* | Haplo | Triple helix | I |
| 33 | F | F | 13 | S/S | 11 | OM | *1A2* | *c.2025+4A>G* | Struct | Triple helix | IV |
| | | M | 51 | 0/0 | | | | | | | |
| 34 | F | F | 26 | C/C | 12/21 | | *1A2* | *c.2746G>A*[a] *p.Gly916Arg* | Struct | Triple helix | I |
| | | F | 41 | M/M | 38/29 | | | | | | |
| | | M | 89 | 0/0 | | | | | | | |
| 35 | F | F | 41 | M/M | 25 | | *1A1* | *c.2073delT p.Gly692ValfsX74* | Haplo | Triple helix | I |
| 36 | F | M | 54 | M/M | 38 | OM; SF | *1A1* | *c.3910C>T p.Gln1304X* | Haplo | C-terminal propeptide | I |
| 37 | F | F | 10 | M/M | 8 | | *1A1* | *c.3100-1G>A*[a,c] | Unknown | Triple helix | I |
| | | M | 24 | 0/S | 22 | | | | | | |
| | | F | 45 | S/S | 37 | | | | | | |
| 38 | F | M | 26 | M/M | 20 | NE | *1A1* | *c.1812delT*[a] *p.Gly605AlafsX161* | Haplo | Triple helix | I |
| | | M | 35 | M/M | 30 | | | | | | |

| Family No. | F/Sp | Individuals | | Hearing characteristics | | | Genotypic characteristics | | | | OI type |
|---|---|---|---|---|---|---|---|---|---|---|---|
| | | M/F | Age | Hearing (L/R) | Onset | Otological history | *COL* | c-notation p-notation | Struct/Haplo | Location | |
| 39 | F | F | 12 | C/C | 5 | OM | *1A1* | *c.1354-12G>A* | Haplo | Triple helix | I |
| | | F | 14 | 0/C | 14 | | | | | | |
| | | F | 30 | M/M | 15/20 | | | | | | |
| | | F | 40 | 0/0 | | | | | | | |
| | | M | 67 | S/S | 45 | | | | | | |
| 40 | F | F | 19 | M/C | 16 | OM | *1A1* | *c.910insGGGCCCC*[a] *p.Arg304GlyfsX7* | Haplo | Triple helix | I |
| | | F | 39 | M/M | 5 | OM; NE | | | | | |
| | | F | 41 | S/S | 31 | | | | | | |
| 41 | F | F | 30 | C/C | 24 | | *1A2* | *c.2746G>C* *p.Gly916Arg* | Struct | Triple helix | IV |
| 42 | F | F | 28 | S/M | 15 | | *1A1* | *c.3540delC*[a] *p.Gly1181AlafsX58* | Haplo | Triple helix | I |
| 43 | F | F | 49 | 0/0 | | OM | *1A2* | *c.838G>A* *p.Gly280Ser* | Struct | Triple helix | IV |
| 44 | F | M | 20 | M/M | 16 | | *1A1* | *c.1299+1G>A* | Unknown | Triple helix | I |
| 45 | F | M | 59 | M/M | 31 | | *1A1* | *c.3027delT* *p.Gly1010ValfsX98* | Haplo | Triple helix | IV |
| 46 | F | M | 50 | M/M | 35 | | *1A1* | *c.3046-1G>T* | Haplo | Triple helix | I |
| 47 | F | F | 24 | 0/C | 23 | | *1A1* | *c.697-2_697-1del* | Unknown | Triple helix | I |
| 48 | F | M | 31 | M/M | 24 | | *1A1* | *c.757C>T* *p.Arg253X* | Haplo | Triple helix | I |
| 49 | F | F | 40 | 0/0 | | | *1A1* | *c.2028+4A>G*[a] | Haplo[b] | Triple helix | I |
| 50 | F | M | 23 | M/M | 18 | | *1A1* | *c.1299+1G>A* | Unknown | Triple helix | I |
| 51 | F | M | 11 | S/S | 11 | OM | *1A1* | *c.671delG* *p.Gly224ValfsX41* | Haplo | Triple helix | I |
| 52 | F | M | 21 | 0/S | 19 | | *1A1* | *c.3925C>T* *p.Gln1309X* | Haplo | C-terminal propeptide | I |
| 53 | F | F | 23 | S/0 | 18 | | *1A1* | *c.672_673delTCinsA* *p.Pro226LeufsX39* | Haplo | Triple helix | IV |
| 54 | F | M | 11 | C/C | 9 | OM | *1A1* | *c.658C>T* *p.Arg220X* | Haplo | Triple helix | I |
| | | F | 43 | M/M | 16 | OM | | | | | |
| 55 | Sp | F | 28 | C/M | 24 | OM | *1A2* | *c.982G>A* *p.Gly328Ser* | Struct | Triple helix | III |
| 56 | Sp | F | 62 | 0/0 | | | *1A2* | *c.486+1G>C*[a] | Struct[d] | Triple helix | I |

| Family No. | F/Sp | Individuals | | Hearing characteristics | | | Genotypic characteristics | | | | OI type |
|---|---|---|---|---|---|---|---|---|---|---|---|
| | | M/F | Age | Hearing (L/R) | Onset | Otological history | *COL* | c-notation p-notation | Struct/Haplo | Location | |
| 57 | Sp | M | 45 | 0/0 | | SF | *1A1* | *c.1065delT*[a] *p.Gly356ValfsX184* | Haplo | Triple helix | I |
| 58 | Sp | F | 26 | C/0 | 24 | OM; SF | *1A2* | *c.1378G>A* *p.Gly460Ser* | Struct | Triple helix | III |
| 59 | Sp | M | 29 | M/M | 17 | NE | *1A1* | *c.879_880del*[a] *p.Glu294LysfsX16* | Haplo | Triple helix | IV |
| 60 | Sp | F | 11 | M/M | 9 | OM | *1A2* | *c.3043G>A* *p.Gly1015Arg* | Struct | Triple helix | IV |
| 61 | Sp | M | 34 | M/M | 9 | | *1A1* | *c.3076C>T* *p.Arg1026X* | Haplo | Triple helix | I |
| 62 | Sp | F | 13 | M/M | 11 | | *1A1* | *c.2921G>C* *p.Gly974Ala* | Struct | Triple helix | IV |
| 63 | Sp | F | 48 | S/M | 16 | | *1A1* | *c.2366delC*[a] *p.Pro789LeufsX318* | Haplo | Triple helix | I |
| 64 | Sp | M | 9 | S/S | 7 | | *1A2* | *c.2432G>C* *p.Gly811Ala* | Struct | Triple helix | I |

OI=osteogensis imperfecta; F=familial OI; Sp=sporadic OI; M=male; F=female; L=left ear; R=right ear; 0=normal hearing; C=conductive hearing loss; M=mixed hearing loss; S=sensorineural hearing loss; OM=otitis media; NE=noise exposure; SF=skull fracture; *COL*=mutated collagen gene; *1A1=COL1A1*; *1A2=COL1A2*; c-notation: notation of the mutation at cDNA level; p-notation: notation of the mutation at protein level; Struct=structurally abnormal type I collagen; Haplo=haploinsufficiency of type I collagen; C-terminal propeptide=mutation located in the carboxy-terminal propeptide of the α-chain; N-terminal propeptide=mutation located in the amino-terminal propeptide of the α-chain.

Nomenclature of mutations and numbering of the *COL1A1* and *COL1A2* genes is based on the wild-type sequences submitted under [GenBank:NG_007400.1] and [GenBank:NG_007405.1], respectively.

[a]Sequence variant that has not been reported before as it was not yet included in the osteogenesis imperfecta variant database (Dalgleish, R: D**atabase of osteogenesis imperfecta and type III collagen mutations.** [http://www.le.ac.uk/genetics/collagen]).

[b]Haploinsufficiency of type I collagen has previously been confirmed by reduced biochemical migration of type I collagen and a positive *COL1A1* null allele test on the proband's cDNA obtained from skin fibroblasts.

[c]Causality of the mutation was verified using the splice site prediction software **Fruitfly** [http://www.fruitfly.org/seq_tools/splice.html] and **Netgene2 server** [http://www.cbs.dtu.dk/services/NetGene]. Sequencing of 95 controls revealed negative outcomes for this sequence variant.

[d]Deviant biochemical elektrophoretic pattern for type I collagen confirmed the synthesis of structurally abnormal type I collagen.
